# Patterns and trends in causes of child and adolescent mortality 2000–2016: setting the scene for child health redesign

**DOI:** 10.1136/bmjgh-2020-004760

**Published:** 2021-03-17

**Authors:** Kathleen L Strong, Jon Pedersen, Emily White Johansson, Bochen Cao, Theresa Diaz, Regina Guthold, Danzhen You, Jennifer Requejo, Li Liu

**Affiliations:** 1 Maternal, Newborn, Child and Adolescent Health and Ageing, World Health Organization, Geneva, Switzerland; 2 Lead Consultant, !Mikro Consulting, Oslo, Norway; 3 Department of Women’s and Children’s Health, Uppsala University, Uppsala, Sweden; 4 Data Analytics and Delivery, World Health Organization, Geneve, Switzerland; 5 Division of Data, Analytics, Planning and Monitoring, UNICEF, New York, New York, USA; 6 Population, Family and Reproductive Health, Johns Hopkins University Bloomberg School of Public Health, Baltimore, Maryland, USA

**Keywords:** child health, epidemiology, public health

## Abstract

The under-5 mortality rate has declined from 93 deaths per 1000 live births in 1990 to 39 per 1000 live births in 2018. This improvement in child survival warrants an examination of age-specific trends and causes of death over time and across regions and an extension of the survival focus to older children and adolescents. We examine patterns and trends in mortality for neonates, postneonatal infants, young children, older children, young adolescents and older adolescents from 2000 to 2016. Levels and trends in causes of death for children and adolescents under 20 years of age are based on United Nations Inter-agency Group for Child Mortality Estimation for all-cause mortality, the Maternal and Child Epidemiology Estimation group for cause of death among children under-5 and WHO Global Health Estimates for 5–19 year-olds. From 2000 to 2016, the proportion of deaths in young children aged 1–4 years declined in most regions while neonatal deaths became over 25% of all deaths under 20 years in all regions and over 50% of all under-5 deaths in all regions except for sub-Saharan Africa which remains the region with the highest under-5 mortality in the world. Although these estimates have great variability at the country level, the overall regional patterns show that mortality in children under the age of 5 is increasingly concentrated in the neonatal period and in some regions, in older adolescents. The leading causes of disease for children under-5 remain preterm birth and infectious diseases, pneumonia, diarrhoea and malaria. For older children and adolescents, injuries become important causes of death as do interpersonal violence and self-harm. Causes of death vary by region.

**Key questions**

**What is already known?**

- Child survival has improved dramatically over the past 20 years but children under-5 still die from preventable childhood diseases and particularly in sub-Saharan Africa and Southern and Eastern Asia.
- The leading causes of under-5 death are premature birth, birth asphyxia and trauma, congenital anomalies, pneumonia, diarrhoea and malaria.
- Deaths from 5 to 19 years are lower than for children under-5 years and at the same time are spread across more cause categories.
- Injuries replace childhood diseases as a leading cause of death for adolescents aged 10–19 years.

**What are the new findings?**

- We examine cause of death by disaggregated age groups and regions to uncover age and region-specific patterns in cause-specific mortality.
- Cause of deaths differs substantially by age and across all regions, with sub-Saharan Africa having the highest mortality overall and from infectious causes.
- The leading causes of older adolescent death also vary across regions with low and middle-income countries in the Eastern Mediterranean region showing high mortality rates from war and police intervention while the Americas have the highest rates from interpersonal violence.

**What do the new findings imply?**

- Identifying the causes of premature death in children and adolescents and where these occur will lead to changes in programmes and policies to support families and communities and continue child survival gains.
- We call attention to causes of death in older children and adolescents to advocate for measures to address these causes and produce healthier children and adolescents now, healthier adults in the future and a healthier future generation.

## Introduction

From 1990 to 2019, the mortality rate per 1000 live births has fallen from 37 to 17 in neonates and from 93 to 38 in under-5s. For older children and adolescents, mortality rates fall from the peak risk among children under-5 through ages 10–14 years followed by an increase in mortality rates among adolescents aged 15–19 years. Across all ages, even as survival has increased, progress has been uneven geographically. Two regions, sub-Saharan Africa and Central and Southern Asia, accounted for more than 80% of the 5.2 million under-5 deaths in 2019. In fact, half of all under-5 deaths occurred in just five countries: India, Nigeria, Pakistan, Ethiopia and the Democratic Republic of the Congo. India and Nigeria alone accounted for about a third.

We set the scene for a child health ‘redesign’ by examining the patterns in cause of death across age and regional groups in a novel way using data from WHO Global Health Estimates (GHE) for 2016. Knowing the common causes of premature death in children and adolescents and where these occur is essential to targeting policies and programmes to best serve families and communities and continue child survival gains, even in the most disadvantaged populations.

We examine distributions in age-specific and cause-specific mortality across the life course from birth to age 19 years, using the age categories in a way that highlights where the most deaths are occurring in the under-5 age category. For the first time, the data are presented as neonates (0–27 days), postneonatal infants (28 days to 11 months) and young children (1–4 years), and then by 5 year age groups to age 19. Older age groups have not been the focus of previous child mortality analysis but action to address the leading causes of disease in these age groups is needed to maintain the survival gains achieved over the past decades for children under-5.

Furthermore, regional differences in the levels and causes of mortality can inform our collective global child and adolescent health response to prevent child deaths and to reach the Sustainable Development Goals (SDGs) (SDG 3.2.1 end preventable deaths of newborns and children under-5 years of age, with all countries aiming to reduce neonatal mortality to at least as low as 12 per 1000 live births and under-5 mortality to at least as low as 25 per 1000 live births) for child survival.^1 2^ For this reason, data are presented by WHO-modified regions (LMICs by WHO region compared with high-income countries (HIC) combined across regions). Our response needs sound and localised evidence to inform interventions to support boys and girls in reaching their full potential. This evidence must be specific to where children live and to critical age periods as they enter adulthood.

## Methods

### All-cause mortality

All-cause mortality estimates for children under-5 years and 5–14 years were generated by the United Nations Inter-agency Group for Child Mortality Estimation (UN-IGME).^3^ All-cause mortality for 15–19 years was obtained from the WHO GHE. The latest available WHO GHE cause-specific mortality estimates for ages over-5 years (2000–2016) defined the time period for the mortality trends presented here. UN-IGME estimates and a detailed description of their methodology have been published elsewhere.^4 5^ Briefly, UN-IGME annually generates estimates for all-cause child mortality by assessing and adjusting all available national data from censuses, surveys and vital registration systems to develop country-specific trends in mortality rates for children under-5 and, more recently, 5–24 years. For countries with complete vital registration systems, data are derived from country reports based on these systems. For countries with incomplete systems, other data sources are also used and reviewed against a set of quality criteria. For each country, all observations meeting inclusion criteria are included in a statistical model that fits a smooth trend curve across observations and extrapolates to a common reference year. This model also adjusts for data errors including systematic or random measurement error associated with different input data sources.

While the UN-IGME uses the probability of dying within an age group as the standard measure, the WHO conventionally uses age-specific mortality rates in the calculation of cause-specific mortality. The child and adolescent mortality estimates produced by these different processes are not directly comparable. We use age-specific rates to present the results of all-cause and cause-specific mortality for this paper. Both UN-IGME and WHO use the UN Population Division’s World Population Prospects (WPP) as the source of population estimates for countries.^6^

### Cause-specific mortality

Cause of death estimates for children under-5 and the population 5–19 years are produced by separate processes. For children under-5, WHO and the Maternal and Child Epidemiology Estimation (MCEE) group prepare estimates of cause of death using 15 different cause categories specific to this age group (table 1). For 5–19 year-olds, the WHO GHE produces cause of death estimates, the most recent being for the period 2000–2016. WHO GHE incorporates MCEE estimates into its analysis to produce a time series consistent with the mortality envelope set by the UN-IGME for under-5s and the UN Population Division’s WPP life tables for the population over 5 years.^6^ We combined these different sources and methodologies to present a consistent time series for levels and trends in mortality for children and adolescents and by region. Cause categories important to older children and adolescents were added to the 15 cause categories used by the MCEE. These included intentional and unintentional injuries split from the injuries combine group as well as maternal conditions (table 1). The result rankings are presented for single causes only. Remainder cause categories (*other non-communicable diseases* and *other infectious diseases and malnutrition*) are not included in the absolute rankings of causes of death by age and region because the range of diseases and conditions included in these categories is non-specific. Nonetheless, the percentage of deaths accounted for by these categories is presented by age and region when it is a substantial percentage of total deaths.

**Table 1:** Cause of death classifications by age group by WHO Global Health Estimates cause name with associated ICD-10 codes. The remainder cause categories (other infectious diseases and malnutrition and other non-communicable diseases) are not included in the estimated top five causes of death presented in this analysis.

	Cause groups	ICD-10 code
WHO Global Health Estimates high-level cause groups	All causes	A00–Y89
	Group I. Communicable, maternal, perinatal and nutritional conditions	A00–B99, D50–D53, D64.9, E00–E02, E40–E64, G00–G09, H65–H66, J00–J22, J85, N30, N34, N390, N70–N73, O00–P96, U04
	Group II. Non-communicable diseases	C00–C97, D00–D48, D55–D64 (exclude D64.9), D65–D89, E03–E34, E65–E88, F01–F99, G10–G98, H00–H61, H68–H93, I00–I99, J30–J84, J86–J98, K00–K92, L00–L98, M00–M99, N00–N28, N31–N32, N35–N64 (exclude N39.0), N75–N98, Q00–Q99
	Group III. Injuries	V01–Y89
Maternal and Child Epidemiology Estimation group causes for children under-5 years	**Cause name**	**ICD-10 code**
	HIV/AIDS	B20–B24
	Diarrhoeal diseases	A00–A09
	Tetanus	A33–A35 037
	Pertussis	A37
	Measles	B05
	Meningitis/encephalitis	A20.3, A32.1, A39.1
	Malaria	B50–B54, P37.3, P37.4 084
	Lower respiratory infections	H65–H66, J00–J22, J85, P23, U04
	Prematurity	P01.0, P01.1, P07, P22, P25–P28, P52, P61.2, P77
	Birth asphyxia and birth trauma*	P01.7–P02.1, P02.4–P02.6, P03, P10–P15, P20–P21, P24, P50, P90–P91
	Sepsis and other infectious conditions of the newborn	P35–P39 (exclude P37.3, P37.4)
	Other infectious causes and malnutrition	Remainder causes from group I
	Congenital anomalies	Q00–Q99
	Other non-communicable diseases	Remainder causes from group II
	Injuries	V01–Y89 (group III)
Older children and adolescents (5–19 years) In addition to the cause categories used for children under-5 years, the following categories from the WHO Global Health Estimates were added for older children and adolescents.	**Cause name**	**ICD-10 code**
	Maternal conditions	O00–O99
	**Unintentional injuries**	V01–X40, X43, X46–59, Y40–Y86, Y88, Y89
	Road injury	V01–V04, V06, V09–V80, V87, V89, V99
	Drowning	W65–W74
	Natural disasters	X33–X39
	Other injuries	Y40–Y86, Y88, Y89
	**Intentional injuries**	X60–Y09, Y35–Y36, Y870, Y871
	Self-harm	X60–X84, Y870
	Interpersonal violence	X85–Y09, Y871
	Collective violence and legal intervention	Y35–Y36

*Also referred to as ‘intrapartum-related complications’.

†Deaths coded to ‘Symptoms, signs and ill-defined conditions’ (R00–R99 in ICD-10) are distributed proportionately to all for neonatal deaths, but exclusively to group I and group II for the postneonatal deaths.

ICD-10, International Classification of Diseases 10th Revision.

Cause of death patterns by region for the years 2000–2016 are presented by six age groups: newborns (0–27 days), postneonatal infants (1–11 months), young children (1–4 years), older children (5–9 years), young adolescents (10–14 years) and older adolescents (15–19 years). Further disaggregation above 11 months is limited by the availability of data at the country and/or regional level to enable meaningful comparisons.

### Cause-specific under-5 mortality

The MCEE estimation process is harmonised with UN-IGME estimates for number of deaths in countries and regions in this age group for the year 2016. The 15 cause categories include all the major causes of neonatal (0–27 days) and postneonatal (1–59 months) deaths and two residual cause categories: other group I (infectious diseases and maternal, perinatal and nutritional conditions) and other group II (non-communicable diseases). The resulting estimates are incorporated into WHO GHE for mortality and burden of disease for all Member States.

A detailed description of the methodology used to develop cause-specific under-5 mortality estimates has been published elsewhere.^7^ Briefly, cause-specific mortality estimates were separately generated for early neonatal (0–6 days), late neonatal (7–27 days) and postneonatal (1–59 months) periods and, within each age group, the estimation approach varied depending on a country’s data availability and under-5 mortality rate (U5MR). For countries with complete vital registration systems, cause of death distributions were directly estimated from the WHO Mortality Database that contains country-reported statistics. For countries with incomplete vital registration systems and low U5MR, cause of death distributions were predicted from a statistical model fit to data from countries with complete vital registration systems. For countries with incomplete systems and high U5MR, cause of death distributions were predicted from a statistical model fit to data assembled from relevant research conducted in high-mortality countries based mainly on verbal autopsy studies. Additional adjustments were made for specific causes of death, such as HIV/AIDS, malaria and measles, as well as to account for conflicts or natural disasters.

### Cause-specific older children and adolescent mortality

Cause-specific mortality estimates for older children and adolescents are based directly on the WHO GHE for all ages over 5 years and for the years 2000–2016. The data sources and methods used to derive these estimates are discussed in detail elsewhere.^8^ Briefly, WHO life tables from 1990 to 2016 are used to generate all-cause mortality ‘envelopes’ for each country, which were recently revised to produce age/sex-specific mortality rates. The WHO life tables use several types of data inputs to generate these mortality estimates including vital registration data, UN WPP, as well as other analyses taking into account deaths from HIV, conflicts and natural disasters. For countries with complete vital registration systems, death registration data reported to the WHO Mortality Database are used after adjusting for completeness. For countries with incomplete systems, age/sex-specific mortality rates are interpolated from the WPP 2017 revision quinquennial life tables that include additional country-specific data on levels of adult mortality, adjusting for deaths from mortality shocks including HIV, conflicts and disasters.^9 10^ The estimation of cause fractions draw on estimates for specific causes by WHO technical programmes and interagency groups, data from sample registration systems for China and India and the Global Burden of Disease study estimates from Institute for Health Metrics and Evaluation (IHME) for other causes where data from countries or WHO are unavailable.^8 11 12^ Finally, the all-cause mortality ‘envelopes’ for each country are used as a basis for attributing country-level estimates for causes of death by age and sex for 2000–2016.

The data are presented for the world and also across WHO regions, slightly modified by removing HICs from the regional designation and creating a group exclusively for these countries called ‘high income’. The regional disaggregation presents the top causes of preventable child death geographically and by World Bank country income groupings, allowing regionally specific causes of death to be uncovered. This disaggregation provides a way of differentiating health issues in low and middle-income countries (LMICs) by region from those in HICs which as a group have very similar mortality and burden disease profiles for these ages (box 1).

Box 1 **List of WHO Member States making up each of the WHO-modified regions.**

**WHO African Region LMICs:** Algeria, Angola, Benin, Botswana, Burkina Faso, Burundi, Cabo Verde, Cameroon, Central African Republic, Chad, Comoros, Congo, Côte d’Ivoire, Democratic Republic of the Congo, Eritrea, Ethiopia, Gabon, Gambia, Ghana, Guinea, Guinea-Bissau, Kenya, Lesotho, Liberia, Madagascar, Malawi, Mali, Mauritania, Mauritius, Mozambique, Namibia, Niger, Nigeria, Rwanda, Sao Tome and Principe, Senegal, Seychelles, Sierra Leone, South Africa, Swaziland, Togo, Uganda, United Republic of Tanzania, Zambia, Zimbabwe.

**WHO American Region LMICs:** Argentina, Belize, Bolivia (Plurinational State of), Brazil, Colombia, Costa Rica, Cuba, Dominica, Dominican Republic, Ecuador, El Salvador, Grenada, Guatemala, Guyana, Haiti, Honduras, Jamaica, Mexico, Nicaragua, Panama, Paraguay, Peru, Saint Lucia, Saint Vincent and the Grenadines, Suriname, Venezuela (Bolivarian Republic of).

**WHO Eastern Mediterranean Region LMICs:** Afghanistan, Djibouti, Egypt, Iran (Islamic Republic of), Iraq, Jordan, Lebanon, Libya, Morocco, Occupied Palestinian Territory, Pakistan, Somalia, South Sudan, Sudan, Syrian Arab Republic, Tunisia, Yemen.

**WHO European Region LMICs:** Albania, Armenia, Azerbaijan, Belarus, Bosnia and Herzegovina, Bulgaria, Georgia, Hungary, Kazakhstan, Kyrgyzstan, Montenegro, Republic of Moldova, Romania, Serbia, Tajikistan, the former Yugoslav Republic of Macedonia, Turkey, Turkmenistan, Ukraine, Uzbekistan.

**WHO Southeast Asian Region LMICs:** Bangladesh, Bhutan, Democratic People’s Republic of Korea, India, Indonesia, Maldives, Myanmar, Nepal, Sri Lanka, Thailand, Timor-Leste.

**WHO Western Pacific Region LMICs:** Cambodia, China, Cook Islands, Fiji, Kiribati, Lao People’s Democratic Republic, Malaysia, Marshall Islands, Micronesia (Federated States of), Mongolia, Nauru, Niue, Palau, Papua New Guinea, Philippines, Samoa, Solomon Islands, Tonga, Tuvalu, Vanuatu, Vietnam.

**High-income countries:** Andorra, Antigua and Barbuda, Australia, Austria, Bahamas, Bahrain, Barbados, Belgium, Brunei Darussalam, Canada, Chile, Croatia, Cyprus, Czechia, Denmark, Equatorial Guinea, Estonia, Finland, France, Germany, Greece, Iceland, Ireland, Israel, Italy, Japan, Kuwait, Latvia, Lithuania, Luxembourg, Malta, Monaco, Netherlands, New Zealand, Norway, Oman, Poland, Portugal, Puerto Rico, Qatar, Republic of Korea, Russian Federation, Saint Kitts and Nevis, San Marino, Saudi Arabia, Singapore, Slovakia, Slovenia, Spain, Sweden, Switzerland, Taiwan, Trinidad and Tobago, United Arab Emirates, UK, USA, Uruguay.

LMICs, low and middle-income countries.

## Results

### Trends in child and adolescent survival

Survival has improved across all age groups and regions since 2000 (table 2A, B). The highest mortality rates occur in the first 28 days of life. Young adolescents (10–14 years) have the lowest mortality rates of all age groups in the life course. Globally, survival gains for neonates (0–27 days) and those over 5 years (older children, young adolescents and older adolescents) were slower than for the other age groups under-5 years (table 2A), representing reductions of 39%, 40%, 13% and 20%, respectively, compared with the 59% and 50% reductions seen in young children (1–4 years) and postneonatal infants (1–11 months) over the same time period. Across the regions, the poor rate of progress is most pronounced for older adolescents living in LMICs in the Americas (0%) and the Eastern Mediterranean (−7%).

**Table 2:** (A) Comparison of mortality rates across the life course from birth to age 19 years for 2000–2016. Global rates presented are central death rates (deaths per 1000 population) and per cent of under-20 population is shown in parenthesis. (B) Comparison of mortality rates across the life course from birth to age 19 years from 2000 to 2016 by modified WHO region. Regional rates presented are central death rates (deaths per 1000 population) and per cent of under-20 population is shown in parenthesis

**A**																												
Global Year			Neonatal (0–27 days)				Postneonatal infants (1–11 months)					Young children (1–4 years)					Older children (5–9 years)					Young adolescents (10–14 years)				Older adolescents (15–19 years)		
2000			31		(32)		26			(24)		6.3			(25)		1.6			(8)		0.8		(4)		1.5	(7)	
2005			26		(33)		21			(24)		5			(23)		1.4			(8)		0.8		(5)		1.3	(8)	
2010			22		(34)		17			(23)		4			(21)		1.2			(8)		0.8		(5)		1.2	(8)	
2016			19		(35)		13			(22)		2.6			(19)		1			(8)		0.7		(6)		1.2	(10)	
% difference 2000–2016			−39				−50					−59					−40					−13				−20		
**B**																												
Neonatal (0–27 days)															Postneonatal infants (28 days to 11 months)													
	African		Americas		Eastern Mediterranean		Europe		Southeast Asia		Western Pacific		High income		African		Americas		Eastern Mediterranean		Europe		Southeast Asia		Western Pacific		High income	
Year	Rate	%	Rate	%	Rate	%	Rate	%	Rate	%	Rate	%	Rate	%	Rate	%	Rate	%	Rate	%	Rate	%	Rate	%	Rate	%	Rate	%
2000	40.9	22	15.9	36	40.2	41	16.8	34	40.6	40	20.3	40	4.3	33	64	28	14	28	26	23	14	26	23	20	11	21	3	18
2005	35.9	24	12.8	35	35.9	41	12.9	36	34.2	41	14.5	41	3.8	34	50	27	11	26	22	22	10	26	19	20	7.8	21	2	18
2010	31.5	26	11.1	29	32.7	45	9.8	38	28.5	42	9.8	38	3.4	35	37	26	9	29	17	21	8	27	14	19	6.9	24	2	18
2016	27.1	28	9.3	34	28.9	44	7.4	38	22.6	45	6.9	36	3	36	28	25	6	21	14	19	5	26	10	18	5	24	2	18
Difference 2000–2016	−34%		−42%		−28%		−56%		−44%		−66%		−30%		−56%		−57%		−46%		−64%		−57%		−55%		−33%	
Young children (1–4 years)															Older children (5–9 years)													
	African		Americas		Eastern Mediterranean		Europe		Southeast Asia		Western Pacific		High income		African		Americas		Eastern Mediterranean		Europe		Southeast Asia		Western Pacific		High income	
Year	Rate	%	Rate	%	Rate	%	Rate	%	Rate	%	Rate	%	Rate	%	Rate	%	Rate	%	Rate	%	Rate	%	Rate	%	Rate	%	Rate	%
2000	18	32	1	13	6	22	2	13	6	22	2	16	1.4	11	5	9	0.5	5	1	6	0.5	6	2	7	0.7	9	0.2	7
2005	13	30	1	12	5	20	1	11	4	20	1	13	0.3	10	4	9	0.4	6	1	6	0.5	6	1	7	0.6	8	0.1	6
2010	9	27	1.2	13	4	19	0.7	10	3	18	0.8	12	0.3	10	3	10	0.7	10	1	6	0.3	5	1	7	0.5	8	0.1	6
2016	7	24	0.7	10	3	17	0.5	10	2	15	0.6	12	0.2	10	2	10	0.3	6	1	7	0.3	6	0.7	7	0.4	10	0.1	7
Difference 2000–2016	−61%		−30%		−50%		−75%		−67%		−70%		−86%		−60%		−40%		0%		−40%		−65		−43%		−50%	
Young adolescents (10–14 years)															Older adolescents (15–19 years)													
	African		Americas		Eastern Mediterranean		Europe		Southeast Asia		Western Pacific		High income		African		Americas		Eastern Mediterranean		Europe		Southeast Asia		Western Pacific		High income	
Year	Rate	%	Rate	%	Rate	%	Rate	%	Rate	%	Rate	%	Rate	%	Rate	%	Rate	%	Rate	%	Rate	%	Rate	%	Rate	%	Rate	%
2000	2	4	0.5	5	0.8	3	0.4	6	1	4	0.4	6	0.2	8	3.7	5	1.2	12	1.4	5	1.1	15	1.6	7	0.7	8	0.5	24
2005	2	4	0.4	6	0.9	4	0.4	6	0.9	5	0.4	6	0.2	8	3	6	1.1	15	1.5	7	0.9	16	1.5	8	0.5	11	0.5	24
2010	1.8	5	0.7	10	0.8	4	0.3	5	0.7	5	0.3	6	0.1	7	2.9	7	1.5	19	1.2	6	0.7	14	1	9	0.5	11	0.4	23
2016	1.5	5	0.4	7	0.9	5	0.3	5	0.5	5	0.3	7	0.1	7	2.6	8	1.2	22	1.3	7	0.6	14	1	11	0.5	11	0.4	23
Difference 2000–2016	−25%		−18%		17%		−38%		−50%		−25%		−33%		−30%		0%		−7%		−45%		−38%		−29%		−20%	

### Neonates (0–27 days)

Neonatal deaths increased as a proportion of all under-20 deaths from 32% in 2000 to 35% in 2016, even with declining mortality rates for the same period (table 2A). The change indicates that neonates represent an increasing proportion of deaths in children and adolescents under 20 years and maybe even higher than estimated here given that fertility rates have also declined over this same period.

**Figure 1:**
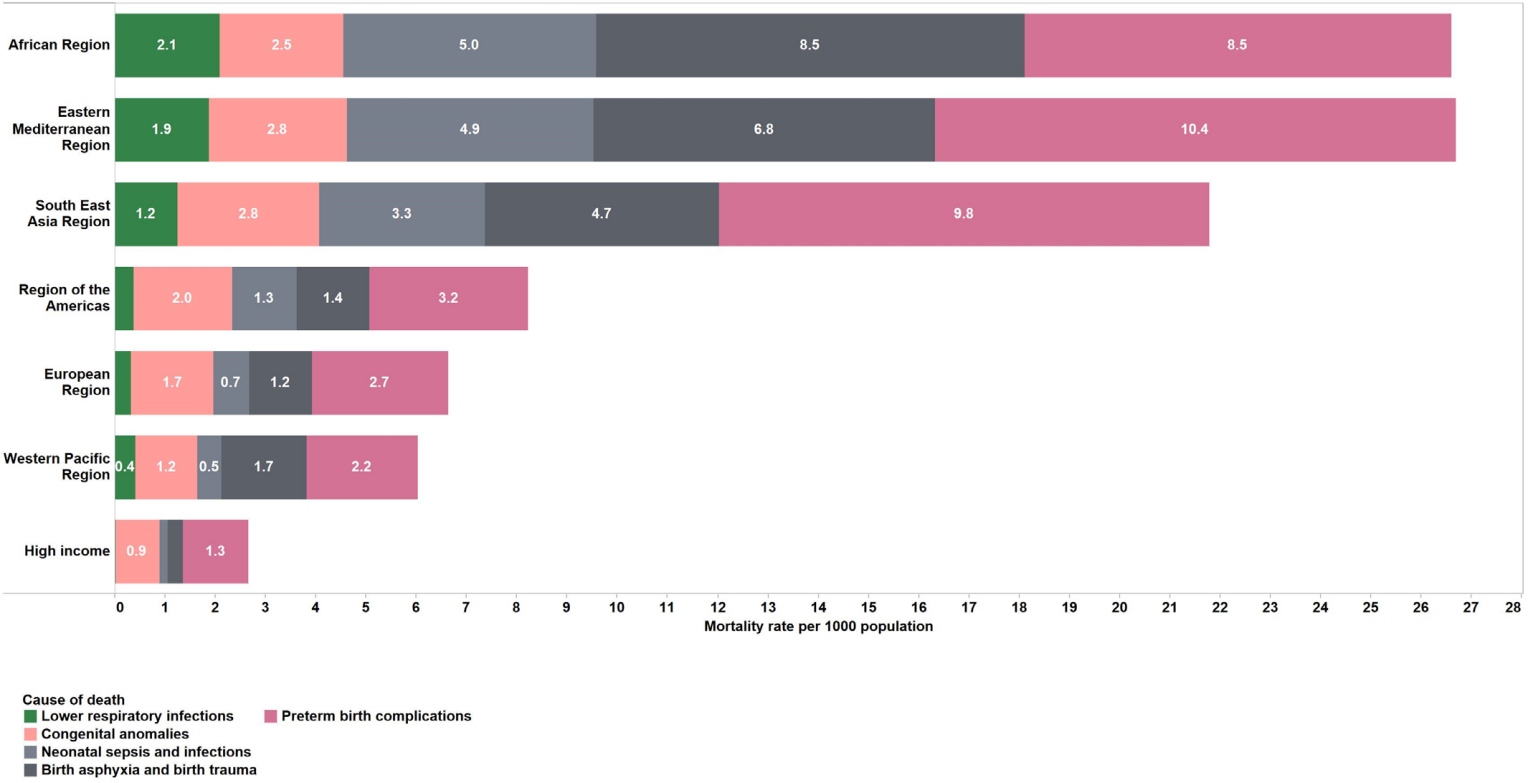
Estimated top five causes of death in neonates (first 28 days of life) by modified WHO region. Numbers in bars are death rates per 1000 population, 2016.

**Figure 2:**
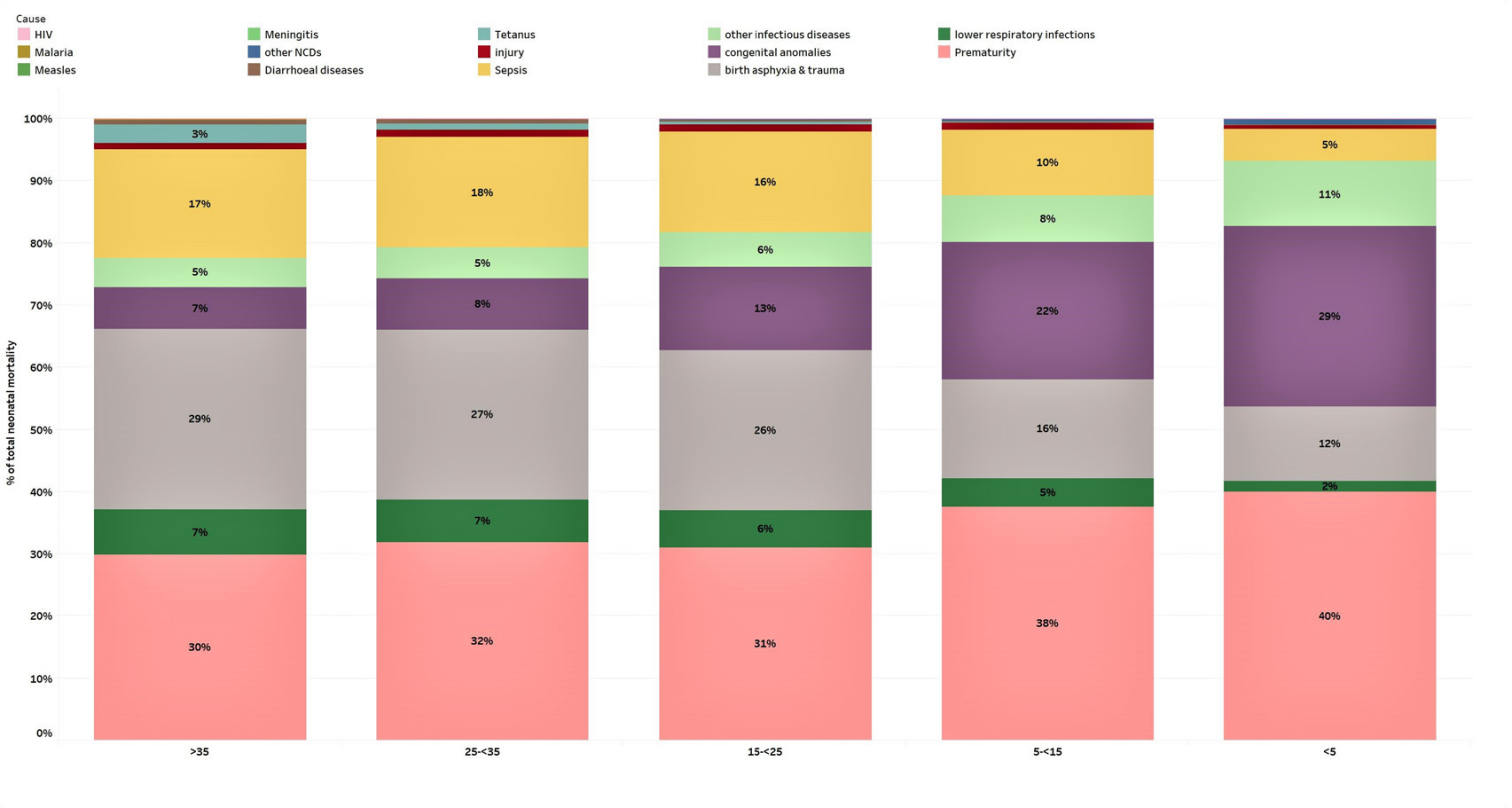
Proportional cause-specific neonatal mortality by categories of neonatal mortality expressed as rates per 1000 live births. Countries with very high neonatal mortality rates (>35 per 1000 live births) are grouped in the far left while countries with very low rates (<5 per 1000 live births) are grouped in the far right. The bars represent the per cent of total neonatal mortality for each cause of death. NCD, non-communicable disease.

Preventable causes of death including preterm birth complications (35% of deaths in this age group), birth asphyxia and trauma (24%), neonatal sepsis and infections (15%) and congenital anomalies (11%) were the top causes of neonatal deaths globally and in most regions in 2016 (table 3A and figure 1). However, an interesting pattern is observed as neonatal mortality declines from greater than 35 deaths per 1000 live births to less than 5; the proportion of deaths caused by birth asphyxia, sepsis and acute respiratory infections also declines leading to an observed increase in the proportion of deaths caused by congenital anomalies and prematurity in the lower mortality groups (figure 2).

**Table 3:** Estimated top five causes of death (rates per 1000 population, numbers (1000s) and per cent of total deaths) for children and adolescents from birth to 19 years of age, globally for 2016. Remainder cause categories (other non-communicable diseases and other infectious diseases and malnutrition) are not included in the rankings. (A) Top five causes of death (estimated rates, numbers and per cent of total deaths) for children under-5 years globally in 2016. (B) Estimated top five causes of death (estimated rates, numbers and per cent of total deaths) for children and adolescents globally in 2016.

	Rate per 1000 population	Estimated number (1000s)	Per cent of total deaths (%)
**A**			
**Neonates aged 0–27 days**			
Preterm birth complications	44	910	35
Birth asphyxia and birth trauma	26	627	24
Neonatal sepsis and infections	17	395	15
Congenital anomalies	16	295	11
Lower respiratory infections	6	159	6
**Postneonatal infants aged 1–11 months**			
Lower respiratory infections	4	465	29
Diarrhoeal diseases	2	257	16
Congenital anomalies	1.2	147	9
Malaria	0.9	110	7
Preterm birth complications	0.7	85	5
**Children aged 1–4 years**			
Lower respiratory infections	0.5	254	18
Diarrhoeal diseases	0.4	204	15
Malaria	0.3	179	13
Injuries	0.2	95	7
Drowning	0.1	55	4
**B**			
**Children aged 5–9 years**			
Diarrhoeal diseases	0.09	60	10
Lower respiratory infections	0.09	60	10
Other injuries	0.08	55	9
Road traffic injury	0.08	49	8
Malaria	0.07	43	7
**Young adolescents aged 10–14 years**			
Other injuries	0.06	40	10
Road traffic injury	0.06	36	9
HIV/AIDS	0.04	27	7
Drowning	0.04	24	6
Lower respiratory infections	0.04	23	6
**Older adolescents aged 15–19 years**			
Road traffic injury	0.17	102	14
Interpersonal violence	0.09	53	8
Self-harm	0.09	53	8
Other injuries	0.09	51	7
Diarrhoeal diseases	0.05	30	4
(Maternal causes) for females	0.05	28	4

### Postneonatal infants (1–11 months)

**Figure 3:**
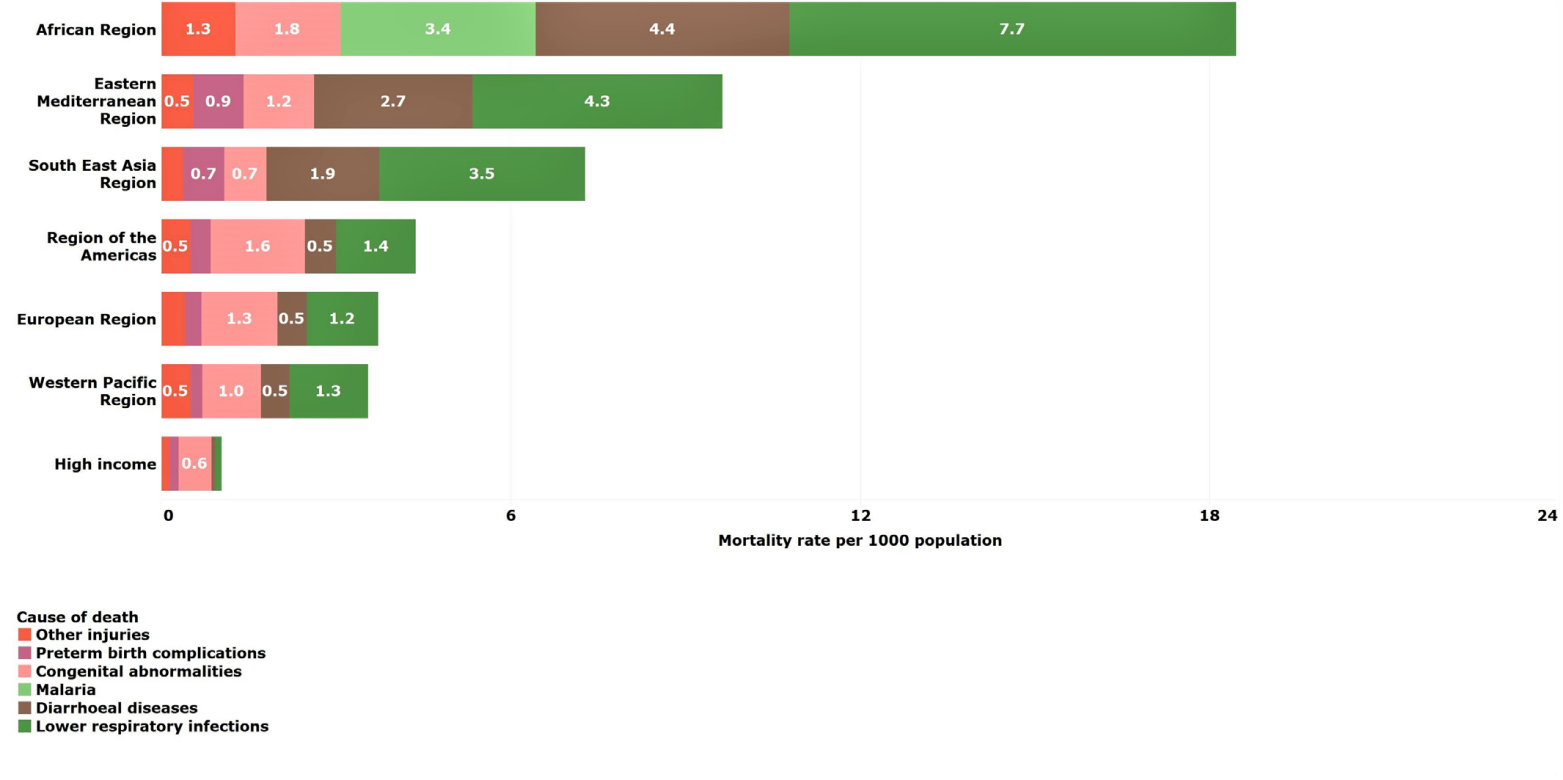
Estimated top five causes of death in postneonatal infants (1–11 months) by modified WHO region. Numbers in bars are death rates per 1000 population, 2016.

Mortality rates for postneonatal infants have declined by 50% from 2000 to 2016. The top five causes of death in this age group were lower respiratory infections (29%), diarrhoeal diseases (16%), congenital anomalies (9%), malaria (7%) and preterm birth conditions (5%). Regional patterns were varied in this age group (table 3A, figure 3). In Africa, Southeast Asia and Eastern Mediterranean LMIC regions, lower respiratory infection and diarrhoea were the leading causes of death. For LMICs in the Western Pacific, European and the Americas regions, congenital anomalies are included along with lower respiratory infections in the top five causes of death. HICs have congenital anomalies, preterm birth complications and other injuries as top causes of death. Other non-communicable diseases make up 25% of deaths in HICs.

### Young children (1–4 years)

**Figure 4:**
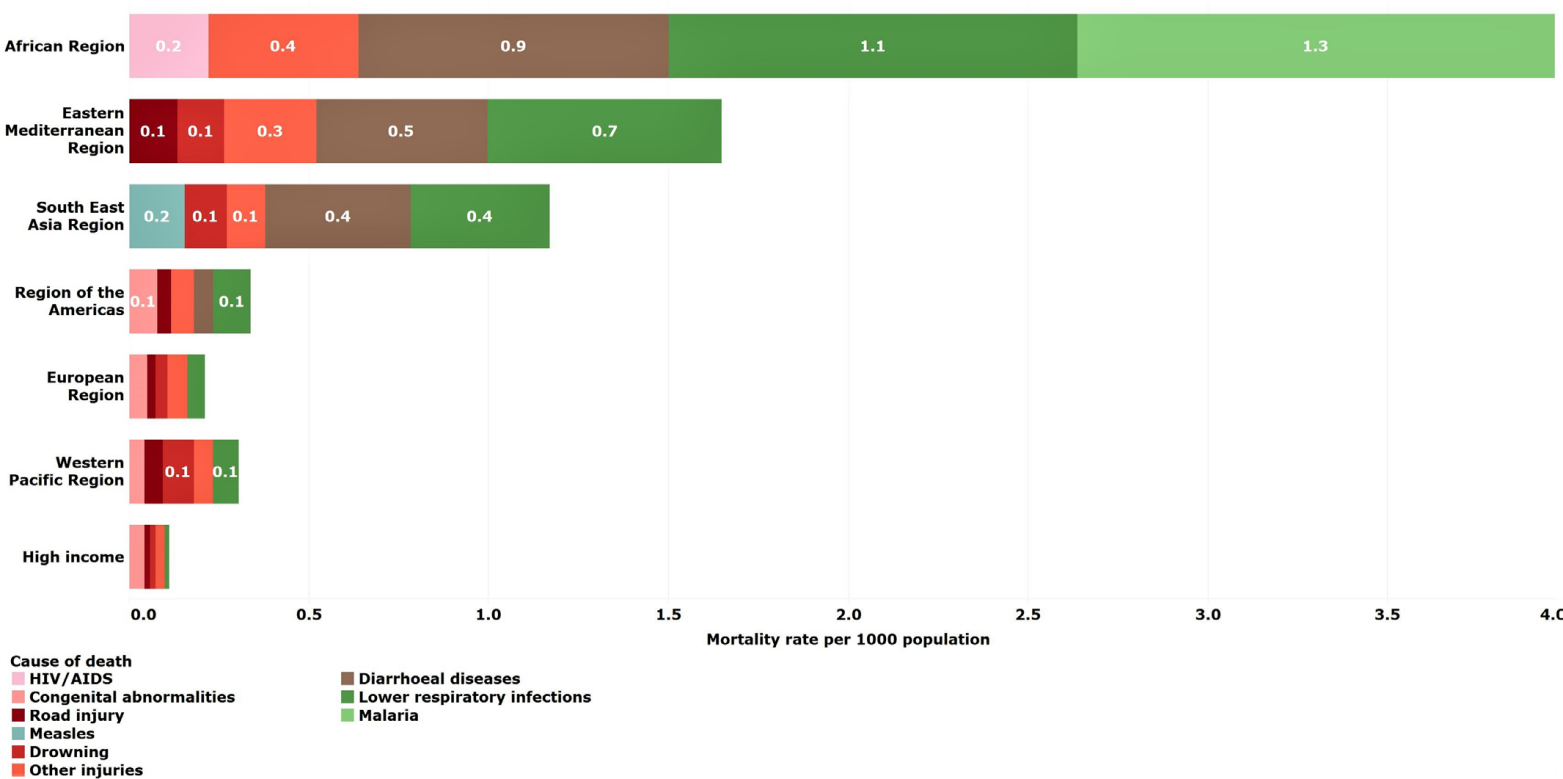
Estimated top five causes of death in young children (1–4 years) by WHO-modified region. Numbers in bars are death rates per 1000 population, 2016.

Reduction in mortality rates was particularly high for young children between 2000 and 2016. All regions except for LMICs in the Americas achieved reductions of 50% or more. Despite these reductions, almost half of deaths among young children were still due to common childhood infections, lower respiratory (18%), diarrhoeal diseases (15%) and malaria (13%), which are both preventable and treatable through simple, affordable interventions (table 3A). The African LMIC region, in particular, remains burdened by these infectious causes of death despite a mortality reduction of 61% from 2000 to 2016 (tables 2B and 3B and figure 4). Other non-communicable diseases are important causes of death for LMICs in Europe (43%) and in HICs (33%).

### Older children (5–9 years)

Globally, mortality rates among older children declined by 40% during this period (table 2A). Every region made progress in reducing older child mortality rates with the largest percentage reductions occurring in Southeast Asian (65%), African (60%) and European (40%) LMICs (table 2B). In fact, the largest absolute reduction in mortality rate among older children occurred in African LMICs—from 4.7 per 1000 in 2000 to 2.5 per 1000 in 2016. The no reduction occurred in Eastern Mediterranean (0%) LMICs presumably due largely to instability caused by regional conflicts.

Globally, the top five causes of death among older children were diarrhoeal diseases (10%), lower respiratory infections (10%), road traffic injuries (8%), malaria (7%) and meningitis (6%) in 2016 (table 3B). Other non-communicable diseases are important causes of death in the Americas (39%), Europe (38%), the Western Pacific (32%) and in HICs (48%).

**Figure 5:**
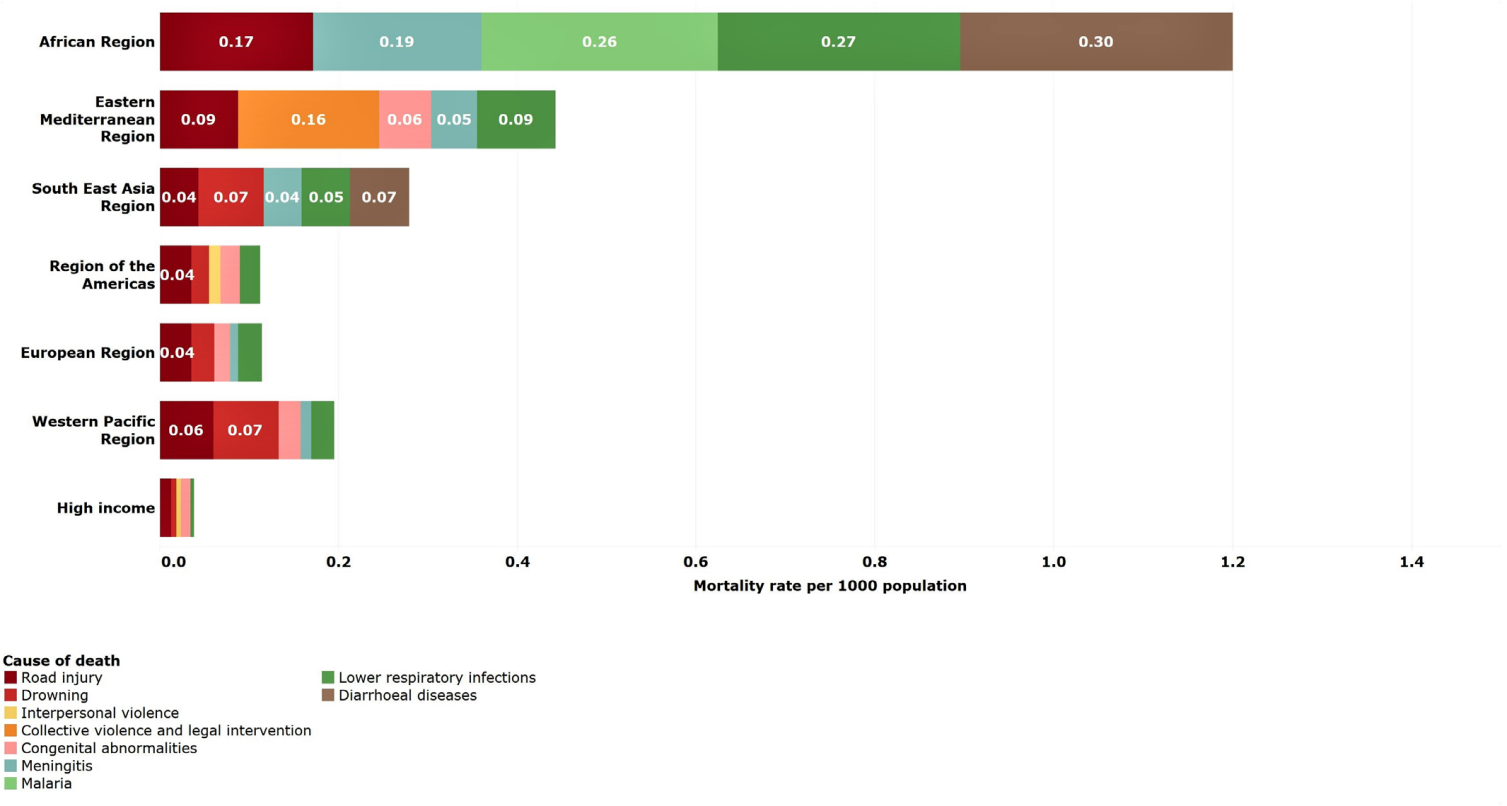
Estimated top five causes of death in older children (5–9 years) by WHO-modified region. Numbers in bars are death rates per 1000 population, 2016.

Despite absolute reductions in mortality rates, African LMICs had substantially higher older child mortality rates compared with all other regions due to the continued burden of infectious diseases including diarrhoeal diseases, lower respiratory infections and malaria (figure 4). Southeast Asian LMICs, in contrast, had the fastest progress of any region. This success was mainly achieved by reducing mortality rates from the same infectious causes that continue to burden the African continent (figure 5).

### Young adolescents (10–14 years)

Global mortality rates for younger adolescents aged 10–14 years were substantially lower than those estimated for older children (5–9 years) and older adolescents (15–19 years). The greatest percentage reductions occurred in Southeast Asian (50%) and European (38%) LMICs. The Eastern Mediterranean LMICs were the only region that experienced rising mortality rates among young adolescents with rates increasing 17% from 2000 to 2016 (table 2B).

**Figure 6:**
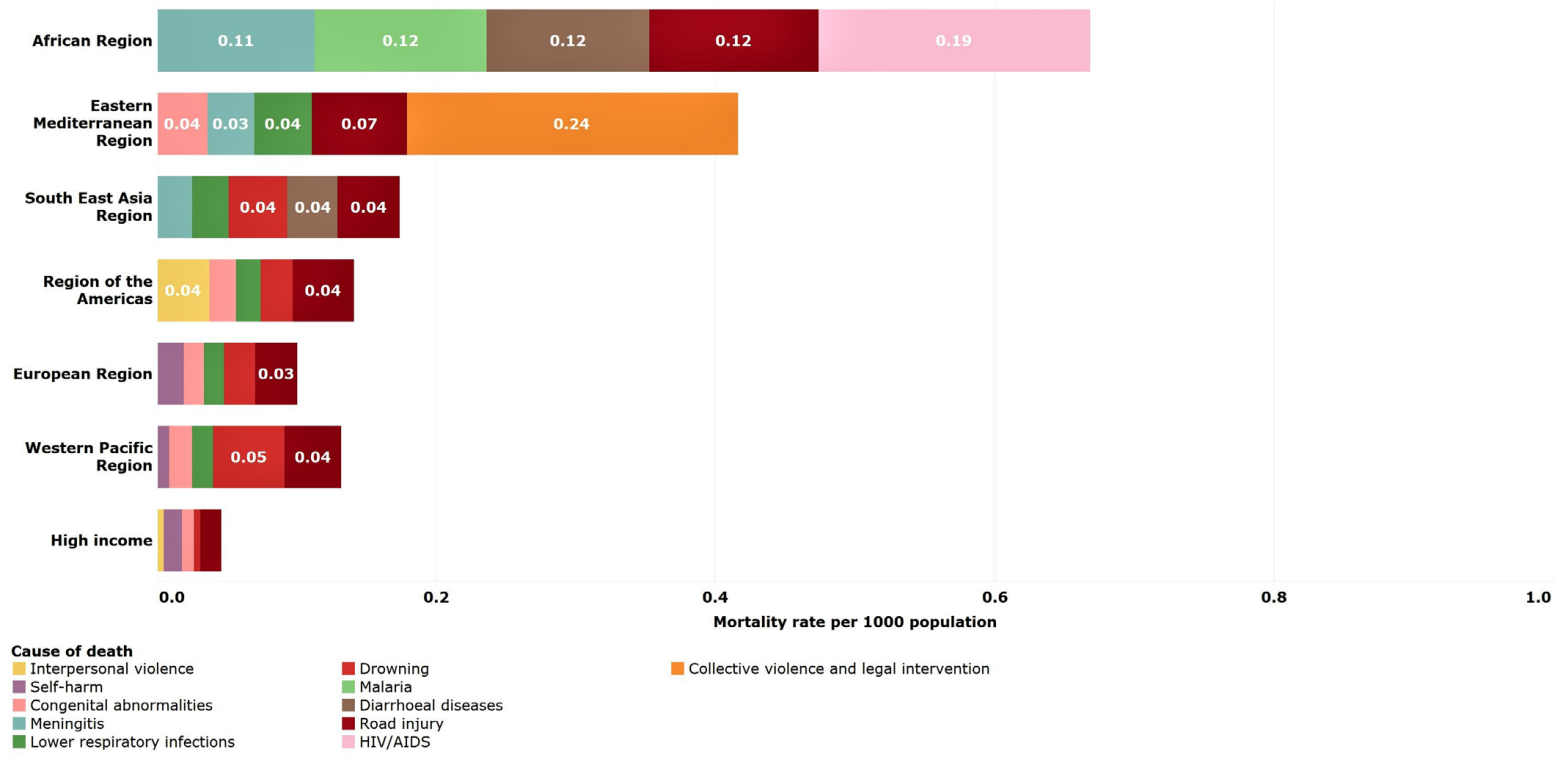
Estimated top five causes of death in young adolescents (10–14 years) by WHO-modified region. Numbers in bars are death rates per 1000 population, 2016.

Road traffic injury (9%) was the leading cause of death globally in 2016 (table 3B). Causes of death were regionally specific with HIV/AIDS as a leading cause in African LMICs, interpersonal violence in LMICs in the Americas and collective violence and legal intervention (war and police intervention) in the Eastern Mediterranean LMICs (figure 6). Across all regions, other non-communicable diseases account for over 20% of deaths in young adolescents.

### Older adolescents (15–19 years)

Global mortality rates among older adolescents declined the least of all age groups under 20 years (20%), except for young adolescents (13%) whose rates are already the lowest across the age span. Older adolescents had a higher death rate compared with older children and young adolescents in each region, especially in the Americas and the Eastern Mediterranean LMIC regions.

**Figure 7:**
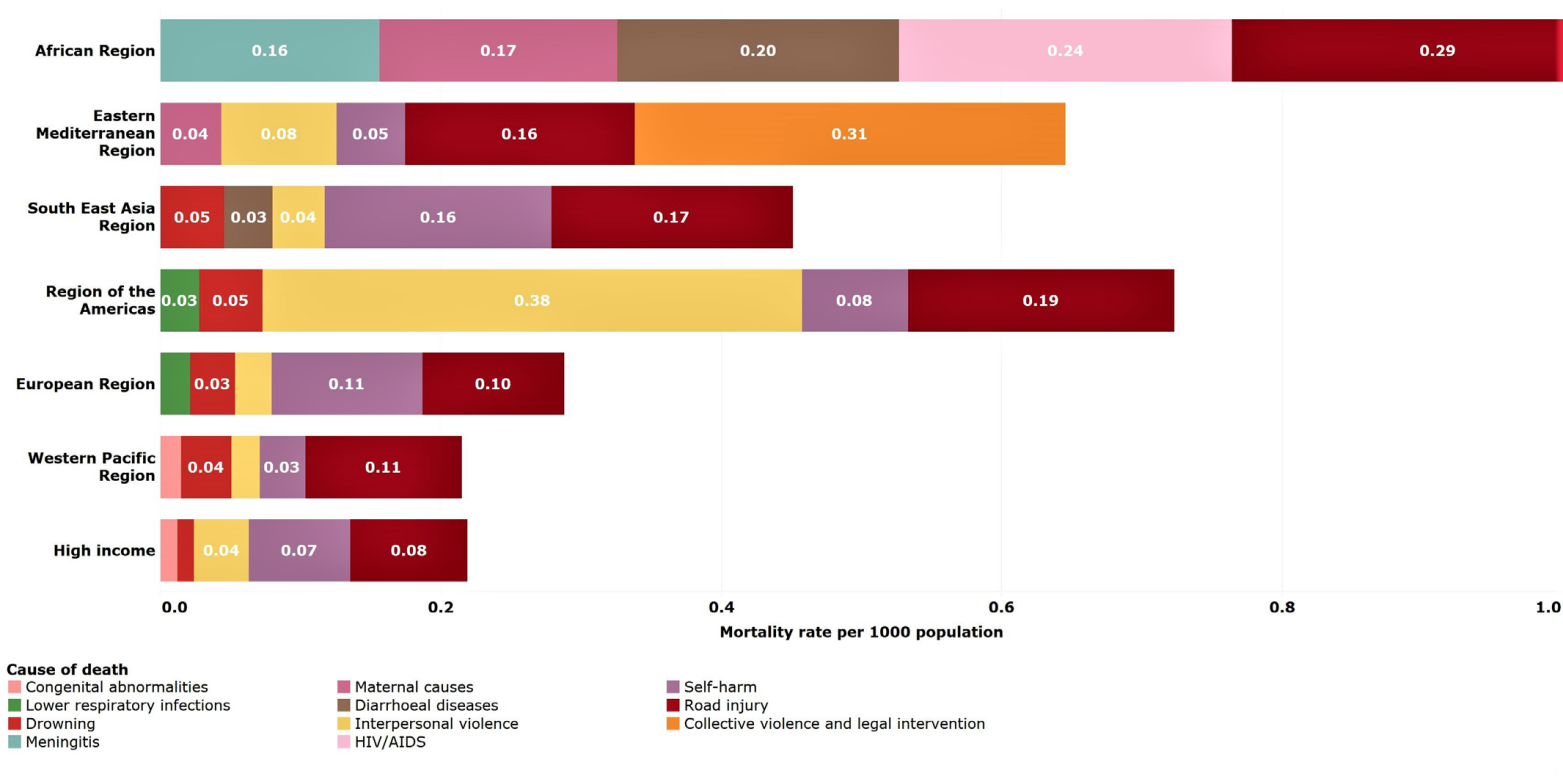
Estimated top five causes of death in young adolescents (15–19 years) by WHO-modified region. Numbers in bars are death rates per 1000 population, 2016.

Leading causes of older adolescent deaths globally are road traffic injury (14%), interpersonal violence (8%), self-harm (8%), diarrhoeal diseases (4%) and maternal causes (4%) (table 3B). As for young adolescents, the leading causes of death are regionally specific, although road traffic injuries are a leading cause in most regions (figure 5). HIV/AIDS, meningitis, maternal conditions and diarrhoea are leading causes in African LMICs, while interpersonal violence is a leading cause of death in LMICs in the Americas. Collective violence and legal intervention (war and police intervention) continues to be a leading cause of death in Eastern Mediterranean LMICs (figure 7). In HICs, other non-communicable diseases account for 49% of all deaths in this age group.

## Discussion

Substantial progress over the past decades in reducing mortality in children and adolescents under-20 years occurred both globally and across regional groupings. Nonetheless, differences between age groups and regions in levels and causes of mortality remain unchanged. For example, sub-Saharan Africa remains the region with the highest mortality rates across all age groups. Most of these deaths are preventable with increased attention to prevention and treatment of common childhood illnesses, including malaria and prevention and treatment of HIV/AIDS.

The reduction in mortality rates from 2000 to 2016 has also been inconsistent across regions. A notable example is the increase in mortality rates for young adolescents in Eastern Mediterranean LMICs. This same region saw a small (7%) reduction in mortality rates for older adolescents, while LMICs in the Americas saw no change at all. These patterns reflect high mortality from interpersonal violence and collective violence and legal intervention in adolescents living in these regions.

### Children under-5 years

Survival for children under-5 has improved over the past two decades; however, important patterns should be addressed to maintain progress in child survival and extend it through adolescence and into young adulthood. Preventing the leading causes of neonatal mortality in LMICs by providing high-quality care for mothers and babies before, during and after birth to prevent birth asphyxia and trauma, prematurity and sepsis is crucial. Accelerated progress for neonatal survival and promotion of health and well-being requires strengthening quality of care for mothers and babies in all regions, and also ensuring availability of quality health services for the small and sick newborns.^13^

In the postneonatal period, lower respiratory infections (mostly pneumonia), diarrhoea and malaria are still the leading causes of preventable deaths. These deaths can be prevented using simple measures including quality antenatal care, breast feeding and proper nutrition for children, access to safe water and food, good hygiene practices, using long-lasting insecticide-treated bed nets to prevent malaria and artemisinin-based combination therapies to treat malaria. Additionally, vaccines are available to prevent the most deadly childhood diseases, including pertussis, diphtheria, tetanus, polio, measles, pneumonia due to *Haemophilus influenzae* type B and *Streptococcus pneumoniae*, and diarrhoea due to rotavirus. Strengthening health systems to provide such interventions to all children will save many more young lives.

### Older children and adolescents

Mortality rates are declining for older children and adolescents but not as fast as for children under-5. Older adolescents face greater mortality risks than younger adolescents and older children given their progression towards sexual development with its associated hormonal changes and increased risk-taking behaviours.^14^ These risks, combined with greater independence and more mobility, result in relatively high rates of preventable deaths from injuries, particularly road traffic injuries and drowning. Preventing these deaths requires multisectoral action from outside of the health domain. Sectors including education, agriculture, transportation and road infrastructure, water and sanitation, and law enforcement have important complementary roles in reducing adolescent mortality. National governments will need to critically assess their countries’ older child and adolescent health needs, determine the most appropriate evidence-based intervention to address them and then prioritise these within their national health programmes, linking to other sectors where appropriate.^15^

Interpersonal violence deaths in older adolescents in LMICs in the Americas are an order of magnitude higher than those of any other region, reflecting a problem with violence that cannot be solved by health systems alone. The destructive power of continued conflict in LMICs in the Eastern Mediterranean is also reflected in the high mortality rates from collective violence and legal intervention in this region. Rates are high for children and adolescents of all ages but older adolescents appear to be disproportionately affected by the violence.

The composite category, other non-communicable diseases, causes between 20% and 49% of deaths for older children, and young and older adolescents across all regions. A recent study describing trends in mortality among older children and young adolescents aged 5–14 years in India, China, Mexico and Brazil reported that cancers are a leading cause of death.^16^ Unpacking the specific conditions that make up this category will be important to future health programming for the older ages.

### Limitations of the study

The general limitations of the WHO GHE 2000–2016 also apply to this paper. They are described elsewhere in detail.^8^ The level of evidence available for mortality estimates for 2000–2016 partly depends on the quality of death registration data available in the WHO Global Mortality Database which is limited for a large number of LMICs, requiring a statistical modelling approach and making the estimates for numbers and causes of death at the country level uncertain. We have not provided uncertainty intervals for these estimates because multiple sources have been combined to produce them. Some sources have uncertainty estimates derived from the source of the data and the modelling process (ie, UN-IGME death estimates for under-5 years) but others are missing uncertainty estimates (ie, WPP estimates for number of births and population size). On reflection, we decided that any uncertainty we could provide would be at best incomplete and at worst misleading. The UN Population Division’s plan to produce probabilistic population projections (from 2019) will partially eliminate this problem and make it easier to produce more meaningful uncertainty estimates in the future.

A more solid empirical base for global estimates of mortality and especially cause-specific mortality will only come from efforts to improve country health information systems, including civil registration and vital statistics (CRVS) systems. Functioning CRVS systems are the key to moving towards timely country-reported information that can improve our knowledge about patterns and trends in levels and causes of mortality. Global progress with CRVS has been slow in the past 30 years but there is evidence that government commitment can lead to substantial progress in the quality of national systems.^17^ Governments that fill this information gap will improve our knowledge of the leading causes of death for children and adolescents in their country while also improving their health outcomes, policies and programmes.^18^ In the meantime, the patterns observed in the time series presented here are similar to those reported from other groups using different data sets and alternative life tables,^11^ ^19^ ^16^ lending credibility to the observed mortality patterns for children and adolescents.

Ending preventable deaths among children and adolescents requires interventions targeted to the age-specific causes of death across different regions. An additional challenge for the global health community in the SDG era is to ensure that the gains made in young child survival are, first, equitably distributed across the world and, second, not lost as children mature into adolescence because their health and development needs are not met.^15^ Indeed, these older age groups are neglected by the international health community which has primarily focused on improving maternal, newborn and young child survival over the past two decades. This means moving beyond the current SDG indicators, which do not have specific indicators to monitor health outcomes for older children and adolescents, and developing better ways to measure health outcomes in these populations on the cusp of adulthood. Achieving the SDG agenda by 2030 will require improved and more systematic monitoring of the health and well-being of the population under-20 years to ensure that all children, everywhere, reach their full potential as adults.

## References

[1] United Nations. Transforming our world: the 2030 agenda for sustainable development. New York: United Nations, 2015.

[2] WHO, UNICEF, UNFPA. Tracking progress towards universal coverage for women’s, children’s and adolescents’ health: the 2017 Report. Washington DC: WHO, UNICEF, UNFPA, 2017.

[3] UNICEF. Un Interagency group on child mortality estimation (UN-IGME). levels and trends in child mortality, report 2019. New York, USA, 2019.

[4] UNICEF. Un Interagency group on child mortality estimation. levels and trends in child mortality report 2019: estimates developed by the un Interagency group for child mortality estimation. NY, USA, 2020.

[5] Alkema L , Chao F , You D , et al. National, regional, and global sex ratios of infant, child, and under-5 mortality and identification of countries with outlying ratios: a systematic assessment. Lancet Glob Health 2014;2:e521–30. 10.1016/S2214-109X(14)70280-3 25304419

[6] World Population Prospects. The 2015 revision. DVD edition. New York: United nations, department of economic and social Affairs, population division, 2015.

[7] WHO. MCEE-WHO methods and data sources for child causes of death 2000-2015. Geneva: World Health Organization, 2016.

[8] WHO. WHO methods and data sources for country-level causes of death 2000-2016. Geneva: World Health Organization, 2018.

[9] UNAIDS. Global AIDS update. Geneva, Switzerland, 2016.

[10] WHO. WHO methods and data sources for life tables 1990-2015. Geneva Switzerland: World Health Organization, 2016.

[11] GBD. 2016 causes of death Collaborators. global, regional, and national age-sex specific mortality for 264 causes of death, 1980– 2016: a systematic analysis for the global burden of disease study 2016. Lancet Global Health 2017;390:1151–210. 10.1016/S0140-6736(17)32152-9 PMC5605883 28919116

[12] UNAIDS. Global AIDS update. Geneva: UNAIDS, 2016.

[13] WHO. Survive and thrive: transforming care for every small and sick newborn. key findings. Geneva: WHO, 2018.

[14] Patton GC , Sawyer SM , Santelli JS , et al. Our future: a Lancet Commission on adolescent health and wellbeing. Lancet 2016;387:2423–78. 10.1016/S0140-6736(16)00579-1 27174304 PMC5832967

[15] WHO. Global accelerated action for the health of adolescents (AA-HA!): guidance to support country implementation. Geneva: World Health Organization, 2017.

[16] Fadel SA , Boschi-Pinto C , Yu S , et al. Trends in cause-specific mortality among children aged 5-14 years from 2005 to 2016 in India, China, Brazil, and Mexico: an analysis of nationally representative mortality studies. Lancet 2019;393:1119–27. 10.1016/S0140-6736(19)30220-X 30876707 PMC6418656

[17] Mikkelsen L , Phillips DE , AbouZahr C , et al. A global assessment of civil registration and vital statistics systems: monitoring data quality and progress. Lancet 2015;386:1395–406. 10.1016/S0140-6736(15)60171-4 25971218

[18] Jackson D , Wenz K , Muniz M , et al. Civil registration and vital statistics in health systems. Bull World Health Organ 2018;96:861–3. 10.2471/BLT.18.213090 30505035 PMC6249696

[19] , Kassebaum N , Kyu HH , et al, Global Burden of Disease Child and Adolescent Health Collaboration. Child and adolescent health from 1990 to 2015: findings from the global burden of diseases, injuries, and risk factors 2015 study. JAMA Pediatr 2017;171:573–92. 10.1001/jamapediatrics.2017.0250 28384795 PMC5540012

